# Epigenetic Regulation of Immune and Inflammatory Responses in Rheumatoid Arthritis

**DOI:** 10.3389/fimmu.2022.881191

**Published:** 2022-04-11

**Authors:** Qi Chen, Hao Li, Yusi Liu, Min Zhao

**Affiliations:** Department of Laboratory Medicine, The First Affiliated Hospital of China Medical University, Shenyang, China

**Keywords:** DNA methylation, rheumatoid arthritis, miRNA, m6A methylation, epigenetic regulation, gene expression

## Abstract

**Purpose:**

Rheumatoid arthritis (RA) is a disease associated with multiple factors. Epigenetics can affect gene expression without altering the DNA sequence. In this study, we aimed to comprehensively analyze epigenetic regulation in RA.

**Methods:**

Using the Gene Expression Omnibus database, we identified a methylation chip, RNA-sequencing, and miRNA microarray for RA. First, we searched for DNA methylation, genes, and miRNAs associated with RA using differential analysis. Second, we determined the regulatory networks for RA-specific methylation, miRNA, and m6A using cross-analysis. Based on these three regulatory networks, we built a comprehensive epigenetic regulatory network and identified hub genes.

**Results:**

Using a differential analysis, we identified 16,852 differentially methylated sites, 4877 differentially expressed genes, and 32 differentially expressed miRNAs. The methylation-expression regulatory network was mainly associated with the PI3K-Akt and T-cell receptor signaling pathways. The miRNA expression regulatory network was mainly related to the MAPK and chemokine signaling pathways. M6A regulatory network was mainly associated with the MAPK signaling pathway. Additionally, five hub genes were identified in the epigenetic regulatory network: *CHD3*, *SETD1B*, *FBXL19*, *SMARCA4*, and *SETD1A*. Functional analysis revealed that these five genes were associated with immune cells and inflammatory responses.

**Conclusion:**

We constructed a comprehensive epigenetic network associated with RA and identified core regulatory genes. This study provides a new direction for future research on the epigenetic mechanisms of RA.

## Introduction

Rheumatoid arthritis (RA) is a chronic autoimmune disease that manifests as an inflammatory change in synovial tissue. It causes erosive joint damage, resulting in impaired articular cartilage and bone, eventually leading to functional disability upon its progression to the terminal phase. RA can also cause fever, anemia, vasculitis, pulmonary interstitial changes, and other systemic injuries (1) thereby increasing the economic burden of treatment. Therefore, determining the cause of RA is urgently needed to reduce the treatment burden and disability rate. While the exact cause of RA is unknown, several factors have been shown to contribute greatly to the pathogenesis of RA, such as genetic factors. First-degree relatives of RA patients (2) have a 2–4 fold increased risk of RA in comparison with the general population, and the concordance rate of RA in monozygotic twins was as high as 15%, fourfold higher than in dizygotic twins due to having the same genetic information (3, 4). At present, a hundred more risk loci for RA have been identified, which helped reveal the pathogenesis of RA and provide new therapeutical targets (5).

Nevertheless, genetic heterogeneity does not explain all the characteristics of RA (6), and there is increasing evidence suggesting that epigenetic modifications play an important role in RA pathogenesis (7). Epigenetics is defined as a heritable but reversible phenomenon that affects gene expression without changing the primary DNA sequence. The main epigenetic phenomenon includes DNA methylation, histone modification, and regulation of non-coding RNAs, such as microRNAs (miRNAs) (8). A previous study reporting a whole-genome DNA methylation analysis in peripheral blood mononuclear cells (PBMCs) of patients with RA suggested that DNA methylation influences the formation of an interferon-inducible gene interaction network associated with RA and highlighted the role of the *PARP9* gene in RA pathogenesis (9). In addition to DNA methylation, miRNAs also play a vital role in the occurrence and progression of RA. MiR-499 is associated with the occurrence of RA, and miR-223 and miR-125b are associated with the development and recurrence of RA and response to treatment (10). RNA methylation is also a form of epigenetic regulation; N6-methyladenosine (m6A), one of the most abundant internal modifications of mRNA in eukaryotic cells, plays a crucial role in many diseases, including cancer (11) and cardiovascular diseases (12). Qing Luo et al. found that decreased expressions of ALKBH5, FTO, and YTHDF2, which are enzymes necessary for the m6A mRNA modification, are risk factors for RA (13). Hui Jiang et al. established a transcriptional map of m6A in MH7A cells and suggested that m6A methylation is possibly associated with the occurrence and progression of RA (14). However, the role of m6A methylation in RA pathogenesis remains unclear.

Although the role of epigenetics in RA has been studied, there has been no comprehensive study on the abnormal expression of various epigenetic mechanisms in RA. Therefore, in this study we analyzed the abnormal expression of DNA methylation-, microRNA, and m6A-related genes by comparing three high-throughput datasets containing the synovial tissues of patients with RA and osteoarthritis to identify the potential regulatory genes of RA.

## Material and Methods

### Data Collection

To analyze the effect of epigenetic regulation on RA, we collected a DNA methylation chip (GSE46364), RNA sequencing (RNA-seq) chip (GSE89408), and miRNA microarray (GSE72564) from the Gene Expression Omnibus (GEO) datasets (https://www.ncbi.nlm.nih.gov/geo/). GSE46364 is an Illumina HumanMethylation450 BeadChip containing synovial tissue samples from five patients with osteoarthritis (OA) and six patients with RA, GSE89408 is an RNA-seq chip containing samples from 22 patients with OA and 152 patients with RA, and GSE72564 is a miRNA expression profiler comprising four samples from patients with OA and four samples from patients with RA.

### Differential Gene Filtering

Differential analysis was used for multi-omics data to identify RA-related genes. ChAMP packages were used to identify differentially methylated genes (DMGs) with *P* < 0.05 considered as the “difference criteria” (15). The Deseq2 package in R was used to search for differentially expressed genes (DEGs) in RNA-seq data (16), and the limma package was applied to analyze the miRNA microarray (17). Benjamini & Hochberg algorithm (18) was used to adjust the P value. The absolute value of Log2 foldchange>1 and *P* < 0.05 were considered as the screening criteria for the expression data.

### Epigenetic Network Construction of Methylation, microRNA, and m6A

Since gene expression regulation by methylation is generally negative, we screened hypermethylated-low expression genes (Hyper-LGs) and hypomethylated-high expression genes (Hypo-HGs) based on differentially methylated sites and DEGs.

To investigate the potential regulatory mechanisms of miRNAs in RA, we first predicted the potential target genes of RA-specific miRNAs using the Starbase database (http://starbase.sysu.edu.cn/) and then performed cross-analysis between predicted target genes and differentially expressed mRNAs. Because of the negative regulatory effect of miRNA on mRNA, we sought low expression mRNA with high expression miRNA and high expression mRNA with low expression miRNA.

Additionally, m6A methylation is mainly regulated by 19 related genes (19). The intersection of these 19 genes and results of RNA-seq differential analysis were used to predict the potential target of related genes using m6a2Target (http://m6a2target.canceromics.org/#/home). Subsequently, the obtained potential targets and DEGs were cross-analyzed to identify key target genes in RA.

### Comprehensive Epigenetic Network Construction and Hub Genes Screening

We cross-analyzed the potential regulatory genes based on the above-mentioned methylation, miRNA, and m6A methylation networks to identify genes that were simultaneously affected by them as RA epigenetic-related genes. The STRING database (string-db.org) was used to analyze the protein-protein interaction (PPI) network between these genes and the molecular complex detection (MCODE) clustering algorithm was used to analyze the protein network and identify hub genes.

### Functional Enrichment Analysis

Gene ontology analysis (GO) is a database for annotating genes and gene products (20). It contains terms in three categories: cellular component (CC), molecular function (MF), and biological process (BP). The Kyoto Encyclopedia of Genes and Genomes (KEGG) is a collection of databases that contains information on genomes, biological pathways, diseases, and drugs (21). We performed GO, and KEGG pathway enrichment analysis of DEGs obtained through analysis using clusterProfiler (22). Benjamini & Hochberg algorithm (18) was used to adjust P value. Adjust P values < 0.05 were considered as significant results.

### Analysis of the Functions of Hub Genes in RA

To understand the functions of hub genes in RA, we used the GSVA algorithm to evaluate the scores of 24 types of immune cells in RA samples. We used Pearson’s correlation analysis to observe the relationship between hub genes and immune cells. Because of the crucial role of the inflammatory response in the occurrence and development of RA, we also investigated the co-expression relationship between hub genes and inflammation-related genes. The specific functions of these hub genes were determined using single-gene gene set enrichment analysis (GSEA). Benjamini & Hochberg’s algorithm was used to adjust the P-value (18). Adjust P values < 0.05 were considered as significant results.

## Results

### mRNA Methylation Network Construction

**Figure 1:**
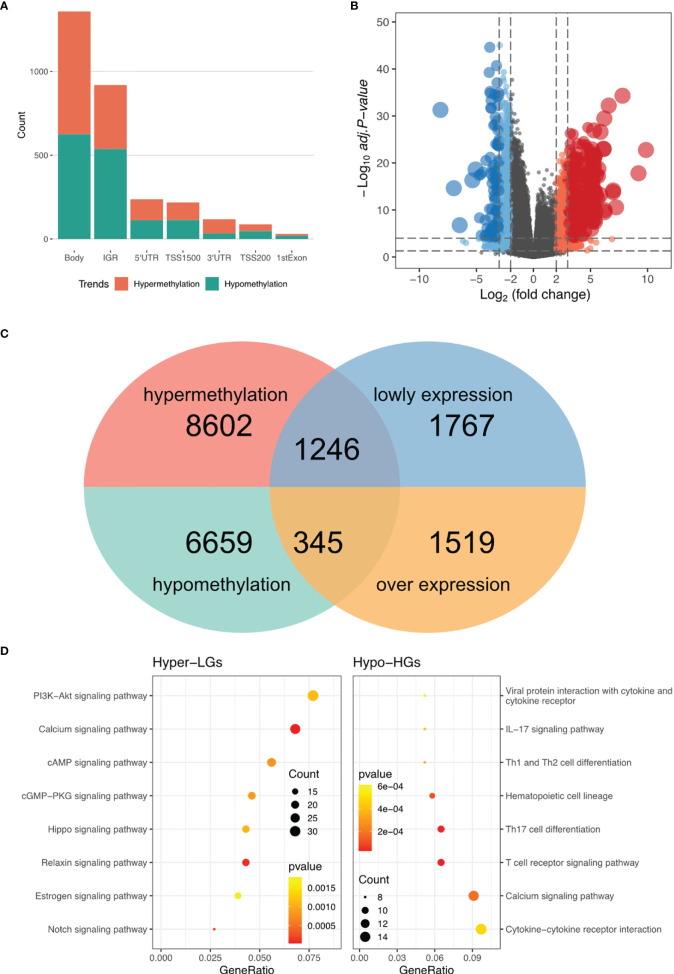
DNA methylation regulatory network in RA. **(A)** Statistic of the genomic location of differentially methylated sites. **(B)** Volcano plot for RNA-seq differential expression analysis. **(C)** Screening of hypermethylated-low expression genes (Hyper-LGs) and highly-expressed genes (Hypo-HGs). **(D)** Kyoto Encyclopedia of Genes and Genomes pathway enrichment analysis of Hyper-LGs and Hypo-HGs.

To construct a methylation network for mRNA methylation in RA, ChAMP was used for differential methylation analysis, and 16,852 differential methylation sites were obtained, including 7,004 hypomethylated sites and 9,848 hypermethylated sites. Based on gene location analysis, we found that both hypermethylated and hypomethylated genes were mainly located in the genomic region and the intergenic region (**Figure 1A**). In addition, differential expression analysis of RNA-seq revealed 1864 highly-expressed genes (Hypo-HGs) and 3013 low-expressed genes (Hyper-LGs) (**Figure 1B**), and 1246 hyper-LGS and 345 hypo-HGs were obtained by cross-analysis (**Figure 1C**). Enrichment analysis of the above genes revealed that hyper-LGs were associated with 25 signaling pathways, including the PI3K-Akt, cAMP, and Hippo signaling pathways. In contrast, hypo-HGs were mainly related to immune processes, including Th1 and Th2 cell differentiation and the T-cell receptor signaling pathway (**Figure 1D** and **Table 1**).

**Table 1:** Functional analysis of DNA methylation regulatory network.

Trends	Term	Description	Count	pvalue
HyperLow	hsa04974	Protein digestion and absorption	21	3.95E-08
	hsa04512	ECM-receptor interaction	19	6.72E-08
	hsa04724	Glutamatergic synapse	21	2.47E-07
	hsa04510	Focal adhesion	28	1.12E-06
	hsa05032	Morphine addiction	16	1.26E-05
	hsa04020	Calcium signaling pathway	28	3.50E-05
	hsa04360	Axon guidance	23	5.09E-05
	hsa04926	Relaxin signaling pathway	18	9.28E-05
	hsa04713	Circadian entrainment	15	0.00011
	hsa01522	Endocrine resistance	15	0.000124
	hsa05033	Nicotine addiction	9	0.000147
	hsa05165	Human papillomavirus infection	33	0.000167
	hsa04330	Notch signaling pathway	11	0.000168
	hsa04727	GABAergic synapse	13	0.000553
	hsa04024	cAMP signaling pathway	23	0.000799
	hsa04022	cGMP-PKG signaling pathway	19	0.000863
	hsa04390	Hippo signaling pathway	18	0.001071
	hsa04151	PI3K-Akt signaling pathway	32	0.001165
	hsa04080	Neuroactive ligand-receptor interaction	31	0.001266
	hsa04915	Estrogen signaling pathway	16	0.001776
	hsa05146	Amoebiasis	13	0.002011
	hsa04921	Oxytocin signaling pathway	17	0.002207
	hsa04010	MAPK signaling pathway	27	0.002223
	hsa05224	Breast cancer	16	0.003394
	hsa04925	Aldosterone synthesis and secretion	12	0.004127
HypoOver	hsa04660	T cell receptor signaling pathway	10	2.64E-05
	hsa04659	Th17 cell differentiation	10	3.67E-05
	hsa05235	PD-L1 expression and PD-1 checkpoint pathway in cancer	9	4.53E-05
	hsa04640	Hematopoietic cell lineage	9	0.000105
	hsa04020	Calcium signaling pathway	14	0.000184
	hsa04658	Th1 and Th2 cell differentiation	8	0.000347
	hsa04657	IL-17 signaling pathway	8	0.000402
	hsa04060	Cytokine-cytokine receptor interaction	15	0.00048
	hsa04061	Viral protein interaction with cytokine and cytokine receptor	8	0.000611

### miRNA-mRNA Regulatory Network Construction

**Figure 2:**
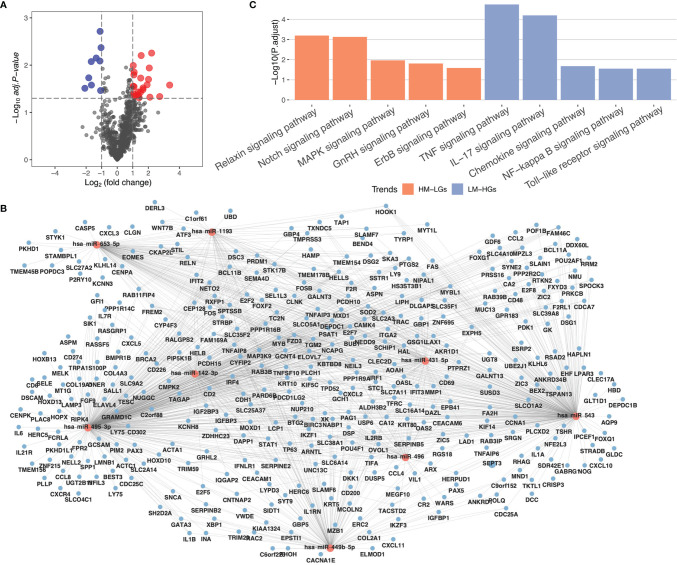
MiRNA regulatory network in RA. **(A)** Volcano plot for miRNA differential expression analysis. **(B)** miRNA regulatory network. Red circles represent miRNAs, and blue circles represent mRNAs. **(C)** KEGG pathway enrichment analysis of highly-expressed miRNAs (HM-LGs) and low-expressed miRNAs (LM-HGs).

To construct a miRNA expression network in RA, differential miRNA analysis was performed using the limma package. A total of 32 differential miRNAs were identified, including nine low-expressed miRNAs and 23 highly-expressed miRNAs (**Figure 2A**). The miRNAs of potential target genes were predicted using the StarBase database and were co-analyzed with DEGs. The results of the co-expression analysis showed that 906 low-expressed genes were regulated by highly-expressed miRNAs (HM-LGs), and 374 highly-expressed genes were regulated by low-expressed miRNAs (LM-HGs) (**Figure 2B** and **Supplementary Table 1**). The pathway enrichment analysis results revealed that HM-LGs were associated with 22 signaling pathways, including the relaxin, Notch, and MAPK signaling pathways. LM-HGs were mainly associated with 17 pathways, including the TNF and chemokine signaling pathways (**Figure 2C** and **Table 2**).

**Table 2:** Functional enrichment analysis of miRNA regulatory networks.

Trends	Term	Description	Count	pvalue
HM-LGs	hsa04510	Focal adhesion	23	2.94E-06
	hsa01522	Endocrine resistance	15	4.86E-06
	hsa04974	Protein digestion and absorption	15	9.11E-06
	hsa04926	Relaxin signaling pathway	17	9.19E-06
	hsa04330	Notch signaling pathway	11	1.34E-05
	hsa04928	Parathyroid hormone synthesis, secretion and action	14	5.55E-05
	hsa04360	Axon guidance	19	7.81E-05
	hsa00532	Glycosaminoglycan biosynthesis - chondroitin sulfate/dermatan sulfate	6	7.99E-05
	hsa04933	AGE-RAGE signaling pathway in diabetic complications	13	0.000120537
	hsa05146	Amoebiasis	13	0.000147866
	hsa04010	MAPK signaling pathway	24	0.000445426
	hsa05165	Human papillomavirus infection	26	0.000469436
	hsa04512	ECM-receptor interaction	11	0.000562502
	hsa04261	Adrenergic signaling in cardiomyocytes	15	0.000696194
	hsa04810	Regulation of actin cytoskeleton	19	0.000798121
	hsa04912	GnRH signaling pathway	11	0.000901842
	hsa04012	ErbB signaling pathway	10	0.001595268
	hsa04935	Growth hormone synthesis, secretion and action	12	0.002181488
	hsa04919	Thyroid hormone signaling pathway	12	0.002511337
	hsa05205	Proteoglycans in cancer	17	0.002537387
	hsa04390	Hippo signaling pathway	14	0.003093187
	hsa04015	Rap1 signaling pathway	17	0.003261983
LM-HGs	hsa04668	TNF signaling pathway	14	7.20E-08
	hsa04657	IL-17 signaling pathway	12	5.09E-07
	hsa04060	Cytokine-cytokine receptor interaction	21	7.74E-07
	hsa04061	Viral protein interaction with cytokine and cytokine receptor	12	1.00E-06
	hsa05143	African trypanosomiasis	6	0.000103648
	hsa05323	Rheumatoid arthritis	9	0.000131484
	hsa04621	NOD-like receptor signaling pathway	13	0.000132753
	hsa04062	Chemokine signaling pathway	12	0.000668445
	hsa05164	Influenza A	11	0.000926237
	hsa04640	Hematopoietic cell lineage	8	0.001050876
	hsa04933	AGE-RAGE signaling pathway in diabetic complications	8	0.001122298
	hsa04064	NF-kappa B signaling pathway	8	0.001447735
	hsa04620	Toll-like receptor signaling pathway	8	0.001447735
	hsa05417	Lipid and atherosclerosis	12	0.001783866
	hsa04659	Th17 cell differentiation	8	0.001844074
	hsa05321	Inflammatory bowel disease	6	0.002274031
	hsa05162	Measles	9	0.00247169

### m6A Regulatory Network Construction

To construct an m6a regulatory network in RA, we analyzed the expression of 19 m6A-related genes mentioned above using differential gene expression analysis, which showed that only *IGF2BP3* was significantly differentially expressed in RA (**Table 3**). The possible target genes of *IGF2BP3* were predicted using the m6A2Target database. They were cross-analyzed with DEGs in RA, which revealed that *IGF2BP3* influences 1419 genes involved in the regulation of m6A methylation. Pathway enrichment analysis revealed that these 1419 genes were mainly associated with eight signaling pathways, including the VEGF, MAPK, and ECM-receptor interaction signaling pathways (**Table 4**).

**Table 3:** Differential analysis of m6a-related proteins.

m6aGene	baseMean	log2FoldChange	lfcSE	stat	pvalue	padj
YTHDC1	1796.161526	-0.028221513	0.080886709	-0.348901735	0.727163082	0.80270679
IGF2BP1	2.697784456	-0.281933868	0.560397819	-0.503095941	0.61489682	0.708631271
IGF2BP2	402.3500668	-0.590021278	0.165311101	-3.569157022	0.000358132	0.001238057
IGF2BP3	105.4914646	1.221619118	0.228126806	5.355000316	8.56E-08	5.92E-07
YTHDF1	959.8377988	0.109755315	0.057790866	1.899181016	0.05754068	0.105592872
YTHDF3	2925.769544	0.255598485	0.084646814	3.019587771	0.002531189	0.007033159
YTHDC2	1840.72859	0.329315265	0.089510793	3.679056484	0.000234098	0.000843939
HNRNPA2B1	7087.424742	-0.034613113	0.065149662	-0.531286151	0.595220501	0.691717891
YTHDF2	1639.946419	0.340595006	0.06620877	5.144258181	2.69E-07	1.71E-06
HNRNPC	6292.814787	0.242423905	0.099017759	2.448287117	0.014353722	0.032137332
RBMX	4378.152403	0.213741124	0.078550519	2.721065726	0.006507182	0.016172884
METTL3	844.7837167	0.087648891	0.061145514	1.433447612	0.15172996	0.235997149
METTL14	558.3295856	0.126012711	0.089093881	1.414381216	0.157250002	0.242919061
WTAP	3321.199154	0.707188731	0.129326528	5.468241852	4.55E-08	3.30E-07
RBM15	1143.633457	-0.024373501	0.069823956	-0.349070755	0.727036191	0.802687218
RBM15B	1687.483233	-0.295446918	0.092183229	-3.204996405	0.001350642	0.004021684
FTO	2155.948843	-0.220745072	0.075130092	-2.938171212	0.003301546	0.008895402
ZC3H13	2756.868741	-0.386453097	0.072532073	-5.328030527	9.93E-08	6.79E-07
ALKBH5	1950.070619	-0.411945241	0.097650023	-4.218588273	2.46E-05	0.000109564

**Table 4:** Functional enrichment of m6a regulatory networks.

ID	Description	Count	pvalue
hsa04370	VEGF signaling pathway	13	2.95E-05
hsa04510	Focal adhesion	27	4.66E-05
hsa05165	Human papillomavirus infection	37	0.00011688
hsa05135	Yersinia infection	19	0.00040802
hsa04512	ECM-receptor interaction	14	0.00058222
hsa04360	Axon guidance	22	0.00101461
hsa04010	MAPK signaling pathway	31	0.00111894
hsa04929	GnRH secretion	11	0.00115637

### Construction of RA-Related Epigenetic Regulatory Network

**Figure 3:**
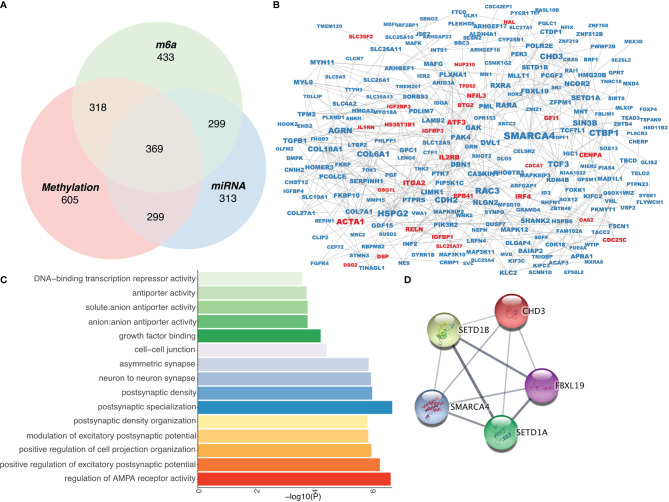
Construction of an epigenetic regulatory network in RA. **(A)** Venn diagram of three epigenetic regulation-related gene screens. **(B)** Protein-protein interaction network of epigenetically regulated genes. **(C)** Gene Ontology analysis of epigenetically regulated genes. **(D)** Interaction network among hub genes.

To identify genes that are simultaneously regulated by the above networks, we cross-analyzed these networks and identified 369 genes (**Figure 3A**). Additionally, PPI analysis revealed 561 edges with an average node degree of 3.46 (**Figure 3B** and **Supplementary Table 2**). GO analysis suggested that these genes were mainly associated with the regulation of AMPA receptor activity, among other pathways (**Figure 3C**). To select PPI hub genes, we used the MCODE plug-in and identified a hub gene module composed of five proteins, including CHD3, SETD1B, FBXL19, SMARCA4, and SETD1A (**Figure 3D**).

### Functional Analysis of Hub Genes

**Figure 4:**
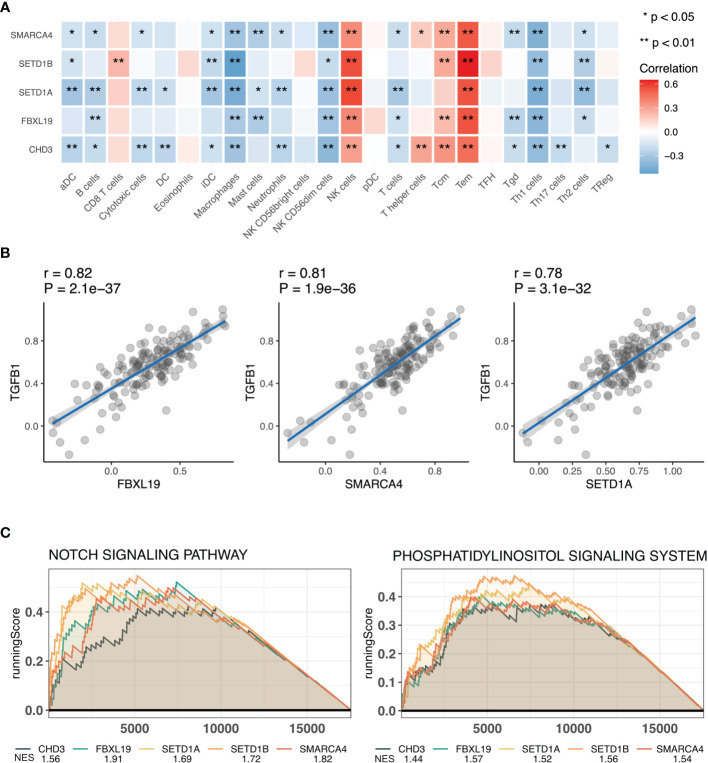
Functional analysis of hub genes. **(A)** Correlation analysis of hub genes and immune cells. **(B)** Scatter plot of correlation analysis of hub genes and transforming growth factor-beta (TGFB1). **(C)** Functional gene-set enrichment analysis (GSEA) of hub genes. *, P<0.05 and **, P<0.01 .

To understand the functions of hub genes, the essential functions of the five obtained genes were analyzed, including their effects on RA immune cells and association with inflammation-related genes. We first calculated the immune cell score of 24 immune cells using GSVA and then compared the correlation between the five genes and immune cells. SETD1A and CHD3 were shown to affect most immune cells, and the five genes were all associated with CD4+ effector memory T (Tem) cells (23) (**Figure 4A**). In addition, because RA is a chronic inflammatory disease, we analyzed the relationship between these five genes and inflammation-related genes. We found that most of the hub genes were associated with inflammation-related genes, of which the transforming growth factor-beta 1 (*TGFB1*) gene and three characteristic genes (*FBXL19*, *SMARCA4*, and *SETD1A*) had a strong positive correlation (**Figure 4B**). Additionally, single-gene GSEA of these five genes revealed that they were all associated with the Notch and phosphatidylinositol signaling pathways (**Figure 4C**). Collectively, these results show that hub genes may influence the occurrence and development of RA through regulating cellular inflammatory responses.

## Discussion

Rheumatoid arthritis is an autoimmune joint disease characterized by irreversible cartilage destruction and bone erosion. The occurrence and progression of RA are greatly influenced by immune (24) and inflammatory responses (25), along with genetic and epigenetic factors. In this study, we aimed to determine the role of epigenetics, including DNA methylation, miRNA, and m6A methylation, in the pathogenesis and development of RA. We comprehensively analyzed these three epigenetic mechanisms to construct an epigenetic regulatory network that is closely associated with immune and inflammatory responses.

We used three high-throughput sequencing assays for a comprehensive epigenetic analysis. In the original article of these data, GSE46364 only analyzed the high-throughput methylation data, and the main functions of RA-related methylation genes were analyzed through enrichment analysis (26). The original article of GSE72564 just selects a suitable miRNA for downstream research. The function of all miRNAs has not been analyzed (27). While GSE89408 mainly analyzed the functions of differentially expressed genes related to RA (28). Comparing the results of our analysis with the previous literature results, the enrichment analysis results of mRNA high-throughput expression data and the results of epigenetic regulatory networks have certain similarities. However, comparing functional analysis of differentially methylated genes with the functional analysis of methylated genes that affect gene expression, we found that there were almost no identical pathways between the two results. Therefore, if we want to analyze methylation sequencing data affected gene regulation function, we still need to combine expression data for cross-analysis.

In the DNA methylation regulation network, the DNA methylation chip and RNA-seq were analyzed to identify differentially methylated and expressed genes. We found 1246 Hyper-LGS and 345 Hypo-HGs associated with RA. The PI3K-Akt signaling pathway plays a crucial role in the cellular inflammatory response (29), as it can affect fibroblast-like synoviocyte metabolism and promote proliferation of synovial cells and osteoclasts (30), which aggravates RA. In our study, Hyper-LGs were enriched with the term “PI3K-Akt signaling pathway”, indicating that in RA, the PI3K signaling pathway is mainly activated *via* DNA methylation. Previous studies have shown that many drugs for the treatment of RA target and inhibit the PI3K signaling pathway (31–33). Therefore, we speculate that drugs that regulate gene methylation may also be candidate drugs for the treatment of RA, providing a potential new target for epigenetic therapy. In addition to the pathways associated with the inflammatory response, we found some immune-related pathways to be significant. Hypo-HGs can affect immune responses, including the T-cell receptor signaling pathway, which affects the development and function of T cells, leading to the occurrence of RA (34–36).

Moreover, we identified 22 pathways associated with highly-expressed miRNAs and 17 pathways associated with low-expressed miRNAs in RA. Most of these pathways were associated with inflammation, including the MAPK (37) and TNF signaling pathways (38). Immune-related pathways, such as the Notch signaling pathway, were also identified in the constructed miRNA-mRNA regulatory network. The Notch signaling pathway not only regulates immune responses but also interferes with osteoclast differentiation, which is involved in bone remodeling (39). In the screening for m6A-related proteins, only IGF2BP3 was found to be related to RA; therefore, we theorized that it was a crucial factor influencing RA-related m6a methylation. IGF2BP3, one of the readers of m6A methylation, has been shown to play a regulatory role in many diseases including cancer (40–42) and cardiovascular diseases (43, 44); however, its role in RA is unknown. In this study, we found that IGF2BP3 is mainly involved in the regulation of inflammation-related pathways, including the MAPK signaling pathway. Additionally, by cross-analyzing these three epigenetic networks, we found that they are all involved in the regulation of inflammatory responses and that miRNA and DNA methylations are also involved in the regulation of immune responses. Our results indicate that epigenetics plays an important role in the regulation of RA.

Because the occurrence of RA is simultaneously regulated by three epigenetic mechanisms. Therefore, we talk about the combination of genes regulated by three mechanisms. Comprehensive analysis to further understand the comprehensive regulation of epigenetics in RA.Based on these three epigenetic networks, we constructed a comprehensive regulatory network in RA and identified 369 epigenetically regulated genes, most of which were expressed at low levels, which is consistent with methylation pattern and miRNA expression. Among these low-expressed genes, SMARCA4 is most associated with other proteins in the entire epigenetic network. SMARCA4 is a member of the SWI/SNF family (45), which have ATPase and helicase activities and regulate gene transcription *via* chromatin remodeling (46). Zhang et al. constructed a neuroendocrine immunomodulation network (NIM) and showed that SMARCA41 plays a critical role in RA (47). Hou et al. revealed that SMARCA4 induces apoptosis of human rheumatoid fibroblast-like synoviocyte MH7A cells in a p53-dependent pattern (48). Therefore, consistent with the results of these previous studies, our study confirms the important role of SMARCA4 in the pathogenesis of RA. Furthermore, we used the MCODE algorithm to identify hub genes in the PPI network, and five genes, *CHD3*, *SETD1B*, *FBXL19*, *SMARCA4*, and *SETD1A*, were selected as hub genes. SETD1A and SETD1B belong to a protein family containing the SET domain and are constituent of a histone methyltransferase (HMT) complex that generated methylated histone H3 at Lys4, indicating that these two proteins are involved in the regulation of methylation. Presently, there are no studies on role of these two genes in RA; therefore, our RA epigenetic network can provide novel mechanistic insights into their functions. CHD3 is a component of the Mi-2/NuRD complex, a type of histone deacetylase complex, which takes part in the of chromatin remodeling by deacetylating histones. FBXL19 can combine with the transmembrane receptor interleukin 1 receptor-like 1 and regulate its ubiquitination and degradation. Zhao et al. showed that FBXL19 inhibits inflammatory response through degradation of the IL-33 receptor, which mediates immune system-related disorders, through ubiquitination (49). The interleukin IL-33 can influence the occurrence of RA. Therefore, FBXL19 can inhibit RA onset by inhibiting the IL-33 receptor (50).

To further understand the role of these genes in RA, we analyzed the relationship between hub genes and immune infiltration and found that all the five genes were associated with multiple immune cells participanting in the pathogenesis of RA, such as Tem cells (51–53) and macrophages (54, 55). Inflammation is the main pathological manifestation of RA, thus, we used *TGFB1* as a representative inflammation-related gene and analyzed its relationship with the five genes. TGFB1, encoding a ligand of the TGF-beta superfamily of proteins, is a central regulator of the inflammatory response. TGFβ-responsive tyrosine phosphatase promotes invasiveness of rheumatoid synovial fibroblast and participates in the pathological mechanism of RA synovial lesions (56). In our study, *FBXL19*, *SMARCA4*, and *SETD1A* showed strong positive correlations with TGFB1 expression. Therefore, these genes play an important role in RA development and should be studied further in future studies.

Our study has several limitations. First, despite mainly using high-throughput sequencing data, the sample size used in our study was relatively small, and the results may be influenced by false positives. Second, we only focused on three epigenetic mechanisms, such as methylation and miRNAs; other epigenetic mechanisms in RA, such as lncRNAs, should be investigated in the future. Finally, this present study is mainly based on high-throughput sequencing, and our results should be clinically validated.

## Conclusion

In conclusion, we performed a comprehensive analysis of epigenetic regulation in RA using public sequencing datasets and determined the main regulatory mechanisms of DNA methylation, RNA methylation, and miRNA expression in RA. Additionally, we constructed a comprehensive epigenetic regulatory network and identified five hub genes, thereby providing new insights into the pathogenesis of RA.

## Data Availability Statement

The datasets presented in this study can be found in online repositories. The names of the repository/repositories and accession number(s) can be found in the article/**Supplementary Material**.

## Author Contributions

QC designed this experiment. QC and HL analyzed the data. QC and YL write the article. MZ reviewed the article. All authors contributed to the article and approved the submitted version.

## Funding

This research was supported by National Key Technologies R&D Program of China provided by Ministry of Science and Technology of the People’s Republic of China (2019YFC0840701) and CAMS Innovation Fund for Medical Sciences (2019-I2M-5-027).

## Conflict of Interest

The authors declare that the research was conducted in the absence of any commercial or financial relationships that could be construed as a potential conflict of interest.

## Publisher’s Note

All claims expressed in this article are solely those of the authors and do not necessarily represent those of their affiliated organizations, or those of the publisher, the editors and the reviewers. Any product that may be evaluated in this article, or claim that may be made by its manufacturer, is not guaranteed or endorsed by the publisher.
